# Concepts on Accumulation of Pesticides and Veterinary Drugs in Fish: A Review with Emphasis in Tilapia

**DOI:** 10.3390/ani13172748

**Published:** 2023-08-29

**Authors:** Claudio Martín Jonsson, Sonia Claudia do Nascimento de Queiroz

**Affiliations:** 1 Laboratório de Aquicultura e Ecotoxicologia, Embrapa Meio Ambiente (Embrapa Environment), Rodovia SP-340, km 127.5, Tanquinho Velho, Jaguariúna 13918-110, SP, Brazil; claudio.jonsson@embrapa.br; 2 Laboratório de Resíduos e Contaminantes, Embrapa Meio Ambiente (Embrapa Environment), Rodovia SP-340, km 127.5, Tanquinho Velho, Jaguariúna 13918-110, SP, Brazil

**Keywords:** contaminants, bioaccumulation, bioconcentration, *BCF*, *Oreochromis niloticus*

## Abstract

**Simple Summary:**

The intensive use of pesticides and veterinary drugs can contaminate aquatic bodies and be accumulated in the organisms that live there. A major concern has been the presence of residues of these substances in some species of fish that are consumed as a source of protein for human beings. Therefore, it is necessary to carry out laboratory studies to find out about the accumulation of these contaminants in the aquatic organism in order to ensure that they are safe for consumption. This review presents the concepts about accumulation of these contaminants in fish, and with this information it is possible to evaluate the risk regarding consumption of this food. More detailed information about studies carried out with tilapia is discussed because it is one of the most consumed fish species in the world.

**Abstract:**

The quality of the aquatic environment can be compromised by the practice of intensive use of pesticides in agriculture and by the misuse of veterinary drugs. Therefore, organisms that live in aquatic ecosystems may be affected due to the presence of these chemicals, through runoff, leaching and other processes. Exposure of aquatic organisms to these xenobiotics could pose health risks. Consequently, there is a growing interest in predicting the bioaccumulation of these substances in aquatic biota from experiments conducted under laboratory conditions. Studies on fish have been performed due to its importance as human food and their wide distribution in most of the aquatic environment. Thus, this article reviews the concepts on determining the accumulation of pesticides and veterinary drugs in fish. The risk regarding the consumption of fish containing residues of these chemical agents, the acceptable daily intake, the testing protocols and the analytical techniques used to determine the residues of these substances in fish tissues are discussed. An emphasis on studies involving tilapia as the test organism was included because, according to Food and Agricultural Organization (FAO), this species is one of the most cultivated in the world.

## 1. Introduction

The reduction in water contamination by pollutants is a global task that must be adopted by developed and developing countries, as claimed by the Food and Agriculture Organization of the United Nations (FAO) [1]. The intensive use of pesticides in agriculture and the misuse of veterinary drugs, among other contaminants, have a high risk of negatively altering the quality of the aquatic environment and compromising the organisms that live there. These facts are due to chemicals losses through runoff, leaching and other processes [2,3]. Accumulation of these organic compounds in aquatic organisms could pose health hazards. Consequently, there is a growing interest in predicting the affinity of these substances for the tissues of aquatic organisms, which can be determined by experiments conducted under laboratory conditions [4]. Thus, this article describes the concepts and methodologies in the accumulation assessment of pesticides and veterinary drugs in fish, since these organisms are important as a protein source for humans and are relevant in the aquatic food chain. 

## 2. The Accumulation Processes of Chemicals in Fish Tissues

Bioaccumulation is a frequent process in aquatic compartments by which pesticides and veterinary drugs are absorbed by organisms directly from the environment (soil, sediment or water) or indirectly by ingestion of food containing these compounds [5]. Compounds must have a certain liposolubility to be retained in body tissues and allow their rate of absorption and storage to be higher than that of excretion [6]. 

Bioaccumulation occurs when a compound’s absorption rate exceeds the compound’s elimination rate, resulting in accumulation in the body. The retention resulting from the intake process of a xenobiotic in an entire organism via water or via air, in aquatic and terrestrial organisms, respectively, is the phenomenon denominated “bioconcentration”. When the compound passes from a lower trophic level to a higher trophic level through the food chain and reaches greater, possibly harmful, concentrations at high trophic levels, this is a “biomagnification” process [7].

Bioconcentration is the most reported process by the literature and the most direct way to evaluate the accumulation of substances in aquatic organisms. It is important to prevent, in tissues of aquatic animals, the presence of contaminants above certain limits. These tissues are used as food for human consumption. The ratio between the concentration of the substance in the organism tissue when apparent equilibrium is reached ($C_{f}$) and the concentration in water ($C_{w}$), is designated as the “bioconcentration factor” (*BCF*). However, this factor can also be calculated by the ratio of the assimilation constant/elimination constant [8]. 

In the assessment of bioconcentration it is important to calculate values of parameters (intake and elimination rate constants, *BCF* and half-life) and compare them with other compounds and other species in addition to associating residues in food with the values of Acceptable Daily Intake (ADI) proposed by the World Health Organization (WHO). Through bioconcentration evaluation tests, it is also possible to study the reduction of the compound in the body’s tissues when exposure ceases and to calculate the residue depletion time to reach levels in the food product that are under current legislation [9].

Chemicals with a high *BCF* are less water soluble and are expected to bioconcentrate in aquatic organisms. Conversely, low *BCF* indicates higher water solubility. In this way, the *BCF* can be estimated by the coefficient of octanol/water partition (K_ow_). The two most commonly used relationships between *BCF* and K_ow_ are “log *BCF* = log K_ow_ − 1.32” and “log *BCF* = 0.79 log K_ow_ − 0.4” [10]. 

According to the EPA (2022) [11], the degree of bioconcentration potential of xenobiotics in fish, as a measure of the *BCF*, can be classified as high, moderate, and low. These categories correspond to *BCF* > 5000, *BCF* = 1000 − 5000, and *BCF* < 1000. 

## 3. Testing Guidelines

Bioaccumulation and bioconcentration testing guidelines provide detailed methods for testing chemicals on fish in controlled laboratory conditions to answer a specific question. This can be evaluating the accumulation potential of a new molecule, comparing the accumulation degree between species, studying a factor that modifies the accumulation, or obtaining data for registration purposes. 

The most described guidelines within the literature are as follows:

OECD (Organization for Economic Co-operation and Development) 305—Bioaccumulation in Fish: Aqueous and Dietary Exposure [12];

ASTM (American Society for Testing and Materials) E1022-22—Standard Guide for Conducting Bioconcentration Tests with Fishes and Saltwater Bivalve Mollusks [13];

EPA (Environmental Protection Agency)—Ecological Effects Test Guidelines OCSPP 850.1730: Fish Bioconcentration Factor (*BCF*) [14].

The OECD 305 method is widely cited by the literature, and there are three different parts within it:

Part I: Aqueous Exposure Bioconcentration Test;

In Part I, which is similarly described in ASTM and EPA methods, the test consists of two phases: the exposure (uptake) and post-exposure (depuration) phases. During the uptake phase, a group of fish of one species is exposed to the test substance at one or more chosen concentrations, depending on the properties of the test substance. They are then transferred to a medium free of the test substance for the depuration phase. A depuration phase is always necessary unless the uptake of the substance during the uptake phase has been insignificant. The concentration of the test substance in/on the fish (or specified tissue thereof) is followed through both phases of the test. In addition to the exposed group, a control group of fish is held under identical conditions except for the absence of the test substance [12,15].

Part II: Minimized Aqueous Exposure Test;

In Part II, a minimized aqueous exposure test is designed in which a reduced number of sample points is possible. The purpose of examining alternative designs for *BCF* studies was to develop a small test to be used in an intermediate testing step to refute or confirm *BCF* estimates based on the octanol/water partition coefficient (K_ow_). This eliminates the need for a full study (as described in Part I) and minimizes cost and animal number, and consequently, reduces the number of tissue and water samples to be analysed [12,16]. 

Part III: Dietary Exposure Bioaccumulation Test.

In Part III, the test consists of an uptake phase (exposure to food spiked with the test-compound) and a depuration phase (exposure to non-spiked food) [12,17]. 

## 4. Determination of the Bioconcentration 

The parameter used to quantify the tendency of a pesticide or veterinary drug to concentrate in fish is the bioconcentration factor (BCF). This corresponds to the relationship between the concentration of the xenobiotic in the organism and its concentration in the environment in which the fish is found, in a state of equilibrium [18].

In environmental and human health risk assessment studies, estimates of predicted or experimentally determined BCF values are fundamental for predicting the accumulation through the trophic chain [19] and to estimate the maximum amount of food intake containing pesticides or veterinary drug residues. The equilibrium or kinetic models can determine this parameter.

### 4.1. Equilibrium Model

When the organism is exposed to a constant concentration of the compound in the water, uptake occurs via branchial respiration (or via the skin or integument), with a gradual increase in tissue concentration as a function of time. The phenomenon occurs with the ingress velocity decreasing until it remains constant. At this moment, called the equilibrium state, the compound’s entry rate is equivalent to the rate of elimination. Under these conditions, the xenobiotic residue in the steady state is directly described by a factor associated with exposure to the environment in which the organism is found [20,21] (Equation (1)).

(1) $$BCF=\frac{C_{f}}{C_{w}}$$

where: 

BCF = bioconcentration factor;

$C_{f}\;$ = concentration of the pesticide or veterinary drug in the fish at steady state;

$C_{w}$ = concentration of the pesticide or veterinary drug in the water.

### 4.2. Kinetic Model

For the calculation of the BCF, the relationship between absorption and elimination, describing the first-order absorption and elimination process in fish, is given by Equation (2) [20,22]:

(2) $$\;\;\;\;\;\;\;\;\;\;\frac{dC_{f}}{dt}=k_{1}C_{w}-k_{2}C_{f}$$

$C_{f}$ = concentration of pesticide or veterinary drugs in fish;
$C_{w}$ = concentration of pesticide or veterinary drugs in water;
$k_{1}$ = rate constant of uptake;
$k_{1}$ = rate constant of elimination;
*t* = time of exposure to water containing the xenobiotic.

When the apparent equilibrium during the accumulation phase of the compound is reached, $dC_{f}/dt=0$, and consequently $k_{1}C_{w}$ = $k_{2}C_{f}$. The BCF is calculated according to the ratio between $C_{f}$ and $C_{w}$ when the steady state is reached [20,23], as was described previously. However, when the steady state is not attained, the ratio between the uptake rate and the elimination rate (Equation (3)) can be used to calculate the BCF.

(3) $$BCF=\frac{C_{f}}{C_{w}}=\frac{k_{1}}{k_{2}}$$

The log of the xenobiotic concentration and the time data of the depuration rate give the elimination constant *k*_2_ by the slope of the regression curve. In order to compute the elimination constant *k*_2_, a multiplication factor (2.303) must be applied because the graph is constructed on a log scale.

(4) $$k_{2}=slope\times 2.303\;$$

The half-life of the xenobiotic (t12b), which is the time with a reduction of 50% in the initial concentration value of the compound, can be calculated as stated by Equation (5) [22]:

(5) t12b=0.693|k2|

When $\;C_{w}$ is constant, Equation (2) can be integrated, resulting in Equation (6) [22,23].

(6) $$\;\;\;\;\;\;\;\;\;\;\;C_{f}=\left(\frac{k_{1}}{k_{2}}\times C_{w}\right)\left(1-e^{-k2t}\right)$$

A graph that relates the compound concentration in fish tissue $\;(C_{f})$ at times (t) of 1.5, 3, 6, 24 and 48 h (for example) of exposure as a function of $1-e^{-k2t}$ could be constructed. The slope of this line is $\frac{k_{1}}{k_{2}}\times C_{w}$. Since $k_{2}$ and *C_w_* are known, it is possible to find the value of $k_{1}$. This last kinetics parameter can also be calculated by Equation (7), where $\frac{\Delta C_{f}}{\Delta t}$ is the tangent of the assimilation curve ($C_{f}$ vs. time) [24].

(7) $$k_{1}=\frac{\left(\frac{\Delta C_{f}}{\Delta t}\right)+k_{2}C_{f}}{C_{w}}\;$$

With both rate constants ($k_{1}$ and $k_{2}$), BCF can be calculated by Equation (3).

The literature describes more sophisticated models to calculate bioconcentration parameters that allow for determining them without an experimental depuration phase [25]. In this way, Andreu-Sanchez et al. [26] investigated the bioconcentration of tebuconazole fungicide in zebrafish (*Danio rerio*) under laboratory conditions and a first-order kinetic pesticide dissipation in the water. The concentrations of tebuconazole fitted to an equivalent nonlinear kinetic-type model, which allowed the calculation of the following parameters: bioconcentration factor (38.80 L kg), time to reach maximum fish concentration (6 days), the maximum concentration in fish (0.0075 μg mg^−1^), half-life in fish (24 days) and time needed for the fish to eliminate 95% of the maximum concentration (105 days). These calculations permitted the establishment of theoretical reference limit values for human consumption of fish and the establishment of safe limits for the water pesticide concentration.

## 5. Analytical Methods

Studies involving the accumulation of xenobiotics in fish and other aquatic organisms have been successfully performed due to the development of modern analytical methods by using appropriated sample preparation and highly sensitive and selective equipment.

Since fish tissue is a complex matrix and the analytes are found in low concentrations (μg kg^−1^ or ng kg^−1^), adequate sample preparation is required. In general, it is necessary to extract the analytes from the matrix, remove the co-extracted endogenous compounds (particularly fat and lipids) and concentrate, if necessary, to adjust the analyte concentration to the instrument’s detector sensitivity.

### 5.1. Extraction Methods

Different types of sample treatment have been used to extract the analytes from the fish tissue. Solid–liquid extraction (SLE) allows soluble components to be removed from solid or semi-solid samples using a solvent [27]. Soxhlet can be used to extract compounds from lipid-containing matrices, but this method involves large amounts of toxic solvent, and it is time-consuming. Microwave-assisted extraction (MAE) [28] and pressurized liquid extraction (PLE) [29] use less solvent and they are considered “greener” extraction methods. MAE uses specific vessels to place the samples and the solvent, which are heated by using microwave energy. The advantages of this method are the reduced extraction time, lower solvent consumption and multiple samples can be simultaneously extracted. However, the high temperature can degrade the thermolabile analytes, special instruments are required and co-extraction of non-target compounds from the matrices can occur. PLE extracts the analytes at a high temperature and pressure and uses a specific instrument. The advantages of this approach are a fully automated process, and it uses less solvent and time to extract the samples. The disadvantages are the same as those cited for MAE.

### 5.2. Clean-Up Procedures

If a clean-up step is not introduced in the sample preparation, the useful life of the chromatographic column, where the separation of the compounds takes place in chromatographic techniques, can be reduced, and the method’s sensitivity can be affected. To remove these interfering compounds and concentrate the analytes, solid-phase extraction (SPE) [30], matrix solid-phase dispersion extraction (MSPD) [31], ultrasound-assisted solid–liquid extraction [32], and gel permeation chromatography (GPC) [33], among others, have been applied. The clean-up using SPE is performed through a column (or cartridge) containing a sorbent, followed by elution with suitable organic solvents and collection of the analytes by fractionation. The sorbents are chosen according to the selectivity, capacity and affinity required. The most popular sorbents for SPE include silica, reverse-phase C18, and strong cation-exchange. The advantages of this type of extraction include small amounts of solvents, a simple application and low cost. MSPD consists of mixing a sample with a solid support, which can be a silica-based material, to obtain a homogeneous mixture. Thus, the mixture is placed in a column or cartridge, as in the SPE, followed by elution with organic solvents of different polarities and elution powers. At the end, the analytes are collected by fractionation. The advantages of this method are as follows: small amounts of samples and solvents, simple application, low cost and simultaneous extraction and clean-up in a single step. The disadvantages are that it cannot be automated and is quite labour intensive. GPC is a useful sample purification technique based on the principle of size exclusion chromatography. It can remove the interferences with high molecular weight, such as natural pigment, grease and steroid hormones.

One of the most used approaches for determination of residues and contaminants in fish is the QuEChERS (Quick, Easy, Cheap, Effective, Rugged and Safe) sample preparation method, which was first introduced by Anastassiades et al. [34]. The QuEChERS was first used to extract pesticides from food matrices such as fruits and vegetables, but nowadays is used, with some modifications, for a wide range of matrices, including fish [9,24]. This method is based on extraction with an organic solvent (mainly acetonitrile) followed by a salting-out step and a dispersive solid-phase extraction (dSPE) clean-up of the resulting organic extracts. QuEChERS offers several advantages over traditional extraction methods, such as high analyte recoveries, accurate results, little use of solvent, fast sample treatment, small lab space, and few equipment requirements. 

Miniaturized extraction techniques that are environmentally friendly have also been used. Some examples are micro-QuEChERS, solid-phase microextraction (SPME) [35], and dispersive liquid–liquid microextraction (DLLME) [36,37]. DLLME has become a very popular environmentally friendly sample preparation technique due to low solvent and reagent consumption, low cost, simplicity and the short time required to perform the extraction. This technique involves the formation of a cloudy solution after rapidly adding a mixture of dispersive (water-miscible solvents) and extractive (water-immiscible solvents) solvents to an aqueous sample. The tiny droplets containing the hydrophobic analytes are separated from the aqueous phase by centrifugation, thus resulting in an enriched extract. Recently, a high-throughput mega-method of sample preparation called “QuEChERSER” (more than QuEChERS) for the analysis of pesticides, veterinary drugs, metabolites, and legacy environmental contaminants in bovine muscles using ultra high-performance liquid chromatography coupled to mass spectrometry (UHPC-MS/MS) and low-pressure (LP) gas chromatography coupled to mass spectrometry (GC-MS/MS) was developed by Monteiro et al. in 2021 [38]. This new mega method has been considered a fast, efficient, and cost-effective analysis and covers a wider polarity range of analytes than QuEChERS. It has been applied to determine multiclass residues of pesticides, veterinary drugs, and environmental contaminants in tilapia muscle [39]. The QuEChERSER method involves many steps, and a more detailed procedure can be obtained directly from the literature [38,39]. 

### 5.3. Instrumental Analysis

Liquid chromatography–tandem mass spectrometry (LC−MS/MS) using different sample preparation methods is the most common technique applied for the multi-residue analyses of pesticides [40] and/or veterinary drugs [41] in fish tissue. For analytes that are not LC-amenable, gas chromatography–tandem mass spectrometry (GC−MS/MS) is often employed [42]. The main advantage of mass spectrometry coupled to chromatographic techniques over many other traditional techniques is the high sensitivity, which allows the unequivocal identification of the analytes (selectivity).

## 6. The Tilapia Fish as a Test Organism to Evaluate the Accumulation of Pesticides and Veterinary Drugs

In the present work, an emphasis on studies involving Nile tilapia (*Oreochromis niloticus*) has been made since this is one of the main species cultivated, according to the Food and Agriculture Organization (FAO) [43]. The main species produced were carp (Grass carp—*Ctenopharyngodon idellus* and Silver carp—*Hypothalmichthys molitrix*) and Nile tilapia [44]. Nile tilapia is widely distributed in the Brazilian territory and created in the most diverse production systems due to its relevant commercial interest [44]. Tilapia represents 51.7% of Brazilian fish production, with 357 thousand tons harvested in 2017, placing Brazil among the top four global farmers [45]. However, few works report on the determination of pesticides/veterinary drugs kinetics parameters in this species to yield data for establishing safety levels in water bodies and tilapia fillets. Table 1 shows the bioconcentration parameters derived from tilapia exposed to 11 pesticides and 1 antibiotic. Pyrethroid insecticides showed the highest BCF values. However, the bioconcentration potential is low (BCF < 1000).

There is a lack of data in the literature about bioaccumulation parameters of veterinary drugs from experiments with tilapia treated with medicated feed. One of these works was conducted by Nunes et al. [9] who exposed tilapias to the antibiotic sulfamethazine in the diet at therapeutic doses. The authors showed that after treatment, the maximum level of sulfamethazine accumulation in the tilapia muscle was 1.6 mg/kg, and the drug was quickly excreted. Thus, considering the acceptable daily intake of sulfamethazine established by the Codex Commission (0–0.05 mg/kg b.w.), and a factor of 5 times the upper amount of fish consumption in Brazil (38 kg/year), the study showed that there is a low risk of adverse effects to consumers.

## 7. The Bioconcentration Factor in the Determination of the Maximum Food Ingestion Quantity

According to the World Health Organization (WHO) [25,49,50], through the expected or actual concentration of a chemical agent in the water, the expected concentration in fish tissue can be estimated when the bioconcentration factor (BCF) is available. The WHO also indicates that an overestimation of the actual fish concentration is likely to be obtained from this calculation. However, the estimate of human exposure by eating fish in the diet can be obtained when we know real or hypothetical concentrations of xenobiotics to which these organisms are exposed.

As stated by the International Life Sciences Institute (ILSI), the acceptable dose intake (ADI) for a xenobiotic is usually compared with the maximum amount of food a person can ingest daily. Therefore, the risk is unacceptable when the ingestion of the compound is bigger than the ADI established [51]. Concerning the assumptions above, Jonsson et al. [25] determined the risk of daily ingestion amount of fillet taken from tilapias which were exposed to herbicide mixtures. The hypothetical concentration of the residue that would be reached in that tissue was calculated. The upper limits of the 95% confidence interval of the BCFs (UL_95%_-BCF) were adopted. The worst-case scenario was adopted to assume the case of “highest risk” by the use of such limits, that is a common practice in risk assessment [46]. This worst situation would be for a person who intakes the fish with the highest pesticide residue, based on the UL_95%_-*BCF*, taken from a water body with the highest herbicide level. This concentration would be attained from the herbicide application directly to the water body surface and to a given water column. The risk of the fish consumption by a population according to the acceptable daily intake (ADI) [25,52] is shown in Table 2. This Table also shows, for each herbicide in each mixture, the estimated maximum concentration of the chemical in the aquatic environment (EMCwater) according to the assumption of Peterson and Hulting [53] and Zagatto [54]. For estimation purposes of the risk, these authors consider a 2 m water column. The application rate (AR) in terms of “kg ha^−1^” described in Table 2 refers to the amount of pesticide applied over a surface area of the water body with the 2 m water column depth.

**Figure 1:**
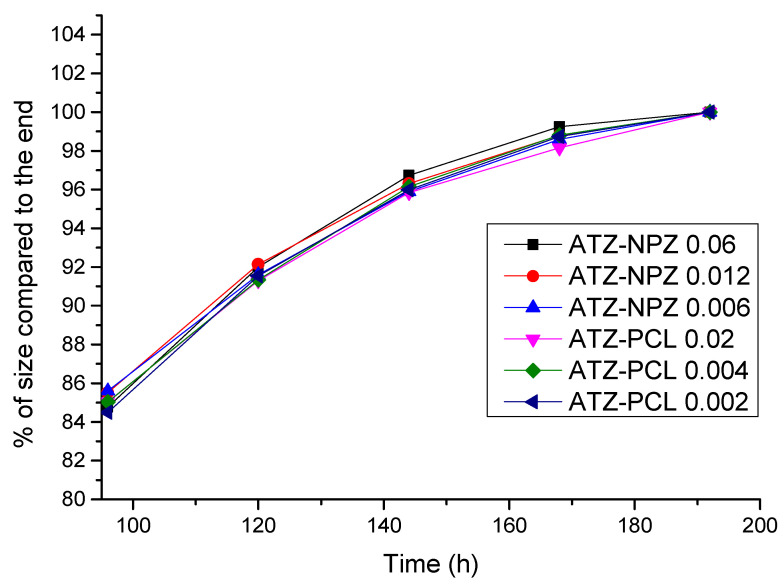
Body growth curves of *Danio rerio* larvae (from 96 to 192 h after egg hatching) fed with microalgae (*Raphidocelis subcapitata*) previously exposed to different concentrations of ATZ-NPZ (atrazine encapsulated in zein nanoparticles) and ATZ-PCL (atrazine encapsulated in poly(epsilon-caprolactone) nanoparticles) (Drawn from Dionisio et al., International Journal of Biological and Natural Sciences 3, DOI 10.22533/at.ed.813342328049, 2023, Atena Publishig Company [55]).

The expected hypothetical concentration of herbicide residue in fish muscle (Exp Cfish) exposed in this situation was calculated by multiplying UL_95%_-BCF by EMCwater. With these data and the ADI value, the maximum daily intake of fish fillet (MDI) may be calculated for an adult person (body weight = 70 kg) in order not to exceed the ADI value (Figure 1), that is, the safe amount of fish fillet to be consumed without risk to human health considering the daily limit of ingestion of the herbicide according to regulatory toxicological parameters. The results in Figure 1 indicate that the greatest restriction is for the herbicide diuron since the individual would need to consume approximately 1.5 kg of fish per day to reach the ADI value.

## 8. Bioaccumulation Studies in the Calculation of the Withdrawal Time (WT)

Withdrawal time (WT) is the minimum time necessary to ensure that the residue of veterinary drug (or pesticide), in a food product, is equal or less than the maximum residue level (MRL).

The study of the accumulation of pharmacologically active substances in edible fish tissues is significant for farmers to manage fish quality properly. Suppose the WT of the veterinary product is not determined for a fish species treated with medicated feed. In that case, there is the potential for the presence of the drug residues in edible tissues in quantities above that which is safe for the consumer [56,57,58]. This risk is due to removing fish for slaughter in a shorter period than necessary for the depletion of residues to a level required by the legislation. 

In this context, it is necessary to evaluate the proper use of veterinary drugs in fish farming, providing a good basis for directing regulatory agencies and stimulating decisions by pharmaceutical companies [56,59,60].

Cordeiro et al. [56] determined the WT for the anthelmintic albendazole in parasitized tambaqui fish (*Colossoma macropomum*) treated with the drug contained in the feed. The fish received a daily therapeutic dose of 10 mg albendazole kg^−1^ body weight (b.w.) via medicated feed (for 34 days) to accumulate the drug. Samples of tissue (muscle plus skin) were collected 24, 48, 72, 120, 168, 240 and 336 h after the end of ABZ administration. The target tissue was stored at −70 °C until analysis by UHPLC-MS/MS for the quantitation of total albendazole residues (albendazole plus metabolites). Considering the tolerance limit of 99% for the percentile population with a confidence limit of 95%, the WT estimate was the time at which the upper unilateral tolerance limit was below the maximum residue level (MRL). The WT was calculated considering the total residue and the MRL value of 100 μg kg^−1^ established by Codex [61].

Thus, considering total analyte residues, an 84 h (approximately four days) WT was estimated for tambaqui parasitized by acanthocephalan and treated with medicated feed. It is important to note that the results were obtained from the experiment at 28 ± 0.5 °C; at lower temperatures, a longer time may be necessary for the elimination of the drug since water temperature influences fish metabolism [56,60].

Therefore, multiplying the WT by the mean of the water temperature (in °C) during the depletion phase means a value refers to “degrees-time”. So, this expression considers the pharmacokinetic profile of the antibiotic, altered by the influence of water temperature on fish metabolism [36,62].

Marques et al. [62] calculated a WT, expressed in “C-days”, equivalent to 129°-days for florfenicol total residues (florfenicol + florfenicol amine) from an experiment conducted with pacu (*Piaractus mesopotamicus*) at 25.8 °C. At this temperature, the WT determined was five days (25.8 × 5 = 129). Therefore, the expression of WT in “degrees-time” can be considered a useful tool for estimating WT when fish are subjected to residue depletion at a different temperature than the normal temperature. Thus, by dividing the value of WT in “degrees-time” by a “new temperature”, it is possible to estimate the “new value” of WT. 

## 9. Bioaccumulation Studies in the Evaluation of the Trophic Transfer Effect

Pesticides and veterinary drugs, accumulated in living organisms, can be transferred through different trophic levels across the food chain. In risk analysis the knowledge about the different exposure routes is important, which is obtained by trophic transfer studies [55,63]. 

Any impact at low levels of the food chain, such as algae which are primary producers, can exert adverse effects at higher trophic levels, such as fishes [55,64]. In this context, Dionisio et al. [55] exposed cultures of the microalgae *Raphidocelis subcapitata* to sublethal concentrations of the herbicide atrazine encapsulated in zein and poly(epsilon-caprolactone) nanoparticles during eight days of growth to bioconcentrate the nanopesticide in algal cells. These organisms were used to feed zebrafish (*Danio rerio*) larvae, in which the growth of the body size was evaluated. 

The results in Figure 1 showed that the atrazine did not alter the larval growth pattern at all the concentrations that the microalgae were exposed. This concentration corresponds to the IC10–96 h (inhibitory concentration at 10% of algal growth for 96 h), 1/5 IC10–96 h, and 1/10 of IC10–96 h to maintain viability and allow microalgae growth with concomitant incorporation of the herbicide in this organism. No statistical growth rate decrease (% length/h) relative to the control (*p* > 0.05) was evidenced for all treatments. Results suggest that the trophic via transfer “via algae” of ATZ in the evaluated nanoformulations would not present a risk for fish development, and this represents an advantage for the potential use and commercialization. 

## 10. Conclusions

Concern about xenobiotics in the environment has in part been spurred by advances in low-level analytical detection of these substances. With the adoption of mass spectrometry (MS) detection coupled with chromatography, very sensitive analytical methods of analysis have gained notoriety for determining the level of residues of different classes of pesticides and veterinary drugs in a variety of sample matrices, including fish. 

Due to the importance of fish as human food and their distribution in the aquatic environment, these animals have been used for bioaccumulation assessments. The availability of standardized testing protocols, contribute to generation of relevant information, and these results help to establish protocols for the safe use of these substances (pesticides and veterinary drugs, among others) and to establish concentration levels in aquaculture to avoid fish residues with risks to humans.

Regarding the large number of compounds used in agriculture, livestock and aquaculture, there are few works that report the determination of kinetic parameters of these chemical agents in fish species, mainly in tilapia—one of the most cultivated fish in the world—to provide data to establish safety levels in water bodies and fish fillets. It is also possible to observe that most of the studies are focused on muscle tissue and rarely on specific tissues, such as bile, liver and plasma. Therefore, more projects of this scope are necessary.

As future prospects, research on climate change, that can alter and modify the distribution and partition of contaminants in water, and studies involving correlation between climate change and bioavailability in aquatic ecosystems with a specific focus on particular contaminants that may pose risks to ecosystems and human health are required. In addition, more research involving nanoencapsulated compounds, which have controlled release, is needed.

## Figures and Tables

**Table 1:** Bioconcentration factors (muscle), kinetics parameters, and exposure conditions on *Oreochromis niloticus* exposed to xenobiotics.

Compound	Water Concentration	Time of Exposure	*k*_1_	*k*_2_	*t*_1/2_	BCF (L·kg^−1^)	Reference
Prometryn	0.055; 0.55 mg L^−1^	28 d	-	-	-	5.76	[15]
Fenpropathrin	2.0 µg L^−1^	48 h	57.73 L·kg^−1^·d^−1^	0.194 d^−1^	3.54 d	295.3	[46]
Cypermethrin	1.5 µg L^−1^	48 h	27.94 L·kg^−1^·d^−1^	0.132 d^−1^	5.24 d	211.2	[46]
Fenvalerate	1.0 µg L^−1^	48 h	54.13 L·kg^−1^·d^−1^	0.168 d^−1^	4.12 d	321.6	[46]
Deltamethrin	1.5 µg L^−1^	48 h	22.31 L·kg^−1^·d^−1^	0.110 d^−1^	6.26 d	201.5	[46]
Ametryn	0.055; 0.296 mg L^−1^	14 d	-	-	-	1.730	[25]
Hexazinone	0.045; 0.473 mg L^−1^	14 d	0.268 L·kg^−1^·d^−1^	0.698 d^−1^	-	0.322	[25]
Diuron	0.109; 1.001 mg L^−1^	14 d	3.742; 6.031 L·kg^−1^·d^−1^	0.840; 1.180 d^−1^	-	4.783	[25]
Tebuthiuron	0.116; 0.955 mg L^−1^	14 d	-	-	-	0.876	[25]
Diclofop-methyl	0.6 µg L^−1^	28 d	-	-	-	2.15	[47]
Lambda-cyhalothrin + acetamiprid	0.066 + 0.0374 µg L^−1^	28 d	-	-	-	29.45–35.41	[48]
Florfenicol	10.00 µg mL^−1^	48 h	0.008 h^−1^	0.15 h^−1^	5 h	0.05	[36]

**Table 2:** Maximum daily intake (MDI) of tilapia fillet from a hypothetical exposure of fish to herbicide mixtures and risk parameters for human consumption. AMT = ametryne; TBUT = tebuthiuron; DIU = diuron; and HEX = hexazinone. UL_95%_-*BCF* = upper limit of the 95% confidence interval of the *BCF*; AR = application rate of the herbicide; EMC_water_ = estimated maximum concentration of the herbicide in the aquatic environment; Exp Cfish = expected hypothetical concentration of the herbicide residue in fish muscle; and ADI = acceptable daily intake of the herbicide. * mg herbicide per kg body weight per day. ** kg fish fillet per day per 70 kg person. (Drawn from Jonsson et al., Heliyon 5, e02237, 2019, Elsevier [25]).

Mixture	Herbicide	${\mathrm{UL}}_{95\%}BCF$	AR (kg ha^−1^)	EMC_water_ (mg L^−1^)	Exp C_fish_ (mg kg^−1^)	ADI *	MDI **
AMT + TBUT	AMT	3.27	2	0.10	0.327	0.02	4.28
	TBUT	1.37	1.2	0.06	0.082	0.07	59.61
(DIU + HEX) +TBUT	HEX	0.51	0.26	0.013	0.007	0.1	1055.81
	DIU	7.10	0.93	0.047	0.334	0.007	1.47
	TBUT	1.44	1.2	0.06	0.086	0.07	56.71
(DIU + HEX) +TBUT + AMT	AMT	1.93	1.5	0.075	0.145	0.02	9.67
	TBUT	1.27	1.2	0.06	0.076	0.07	64.30
	HEX	0.21	0.26	0.013	0.003	0.1	2564.10
	DIU	3.45	0.93	0.047	0.161	0.007	3.02

## Data Availability

Not applicable.
